# Structure-function relationship of an Urokinase Receptor-derived peptide which inhibits the Formyl Peptide Receptor type 1 activity

**DOI:** 10.1038/s41598-019-47900-3

**Published:** 2019-08-21

**Authors:** Michele Minopoli, Andrea Polo, Concetta Ragone, Vincenzo Ingangi, Gennaro Ciliberto, Antonello Pessi, Sabrina Sarno, Alfredo Budillon, Susan Costantini, Maria Vincenza Carriero

**Affiliations:** 1 Neoplastic Progression Unit, Istituto Nazionale Tumori IRCCS “Fondazione G. Pascale”, Naples, Italy; 2 Experimental Pharmacology Unit, Istituto Nazionale Tumori IRCCS “Fondazione G. Pascale”, Napoli, Italy; 3 Immunologia e Diagnostica molecolare Istituto Oncologico Veneto, Padova, Italy; 4 Scientific Directorate, Istituto Nazionale Tumori “Regina Elena”, IRCCS, Roma, Italy; 5 Peptipharma, Roma, Italy

**Keywords:** Computational biology and bioinformatics, Computational models

## Abstract

The interaction between the short 88Ser-Arg-Ser-Arg-Tyr92 sequence of the urokinase receptor (uPAR) and the formyl peptide receptor type 1 (FPR1) elicits cell migration. We generated the Ac-(D)-Tyr-(D)-Arg-Aib-(D)-Arg-NH2 (RI-3) peptide which inhibits the uPAR/FPR1 interaction, reducing migration of FPR1 expressing cells toward N-formyl-methionyl-leucyl-phenylalanine (fMLF) and Ser-Arg-Ser-Arg-Tyr (SRSRY) peptides. To understand the structural basis of the RI-3 inhibitory effects, the FPR1/fMLF, FPR1/SRSRY and FPR1/RI-3 complexes were modeled and analyzed, focusing on the binding pocket of FPR1 and the interaction between the amino acids that signal to the FPR1 C-terminal loop. We found that RI-3 shares the same binding site of fMLF and SRSRY on FPR1. However, while fMLF and SRSRY display the same agonist activation signature (i.e. the series of contacts that transmit the conformational transition throughout the complex), translating binding into signaling, RI-3 does not interact with the activation region of FPR1 and hence does not activate signaling. Indeed, fluorescein-conjugated RI-3 prevents either fMLF and SRSRY uptake on FPR1 without triggering FPR1 internalization and cell motility in the absence of any stimulus. Collectively, our data show that RI-3 is a true FPR1 antagonist and suggest a pharmacophore model useful for development of compounds that selectively inhibit the uPAR-triggered, FPR1-mediated cell migration.

## Introduction

Cell migration is a sequential and interrelated multistep process that regulates physiological processes such as embryonic development, tissue repair and immune-cell trafficking in both embryo and adult tissues^1^. When cell migration is deregulated, it contributes to a variety of pathologic conditions which include chronic inflammation, vascular diseases, and tumor metastasis^2,3^. For this reason, the control of cell motility may be useful for developing new therapeutic strategies aimed to control diseases driven by aberrant cell motility, such as the metastatic dissemination of cancer cells.

The Urokinase-type Plasminogen Activator (uPA) Receptor (uPAR) is a widely recognized master regulator of cell migration^4^. Besides focusing proteolytic activity of uPA on the cell membrane^5^, uPAR, upon binding to uPA, initiates in a protease independent manner the intracellular signaling pathways that regulate physiologic processes such as wound repair and immune responses, as well as pathologic conditions such as inflammation and tumor metastases^4,6^.

uPAR is formed by three domains (DI, DII, and DIII) linked by short sequence regions, anchored to the cell surface by a glycosyl-phosphatidylinositol (GPI) tail. The three domains pack together into a concave structure that binds uPA^7–9^. In the uPAR/uPA complexes, the domain boundary between DI and DII (uPAR84-95 sequence) is mostly projected on the external uPAR surface, comprises a protease-sensitive signaling region and exhibits a structural flexibility in both membrane-associated and soluble forms of uPAR^10,11^.

We and others documented that the uPAR84-95 sequence as well as the synthetic shorter pentapeptide uPAR88-92(Ser-Arg-Ser-Arg-Tyr, SRSRY) elicit chemotaxis and promote directional cell migration and angiogenesis *in vitro* and *in vivo*^12–14^. We proven that both uPAR84-95 and SRSRY exert chemotactic activity by interacting with the formyl peptide receptor type 1 (FPR1) which, in turn, internalizes. After internalization, FPR1 triggers vitronectin receptor activation with an inside-out type of mechanism that involves PKC, AKT and MAPK^13,14^.

Using SRSRY peptide analogues, we found that the Arg89-Ser-Arg91 central core is indispensable for the SRSRY-dependent cell signaling^15^, and identified Ser90 as a key residue within a critical “hinge”, where it affects the conformation of neighboring residues. Substitution of the Ser90 with a glutamic acid residue, in the membrane-associated uPAR, generated a dominant-negative protein that prevents agonist-triggered FPR1 internalization, migration and invasion of sarcoma cells^16^. Interestingly, a cyclized form of the SRSRY peptide elicits opposite effects on cell migration. The cyclic [SRSRY] peptide competes with both SRSRY and N-formylmethionyl-leucyl-phenylalanine (fMLF) peptides for binding to FPR1 and inhibits FPR1activation and internalization, causing a dramatic reduction of monocyte recruitment into inflamedtissues^17–19^.

Originally identified on the surface of myeloid cells as the high-affinity receptor for the fMLF peptide, FPR1 recognizes many and diverse ligands, ranging from formylated peptides derived from bacterial protein degradation and from mitochondrial proteins of eukaryotic cells, to non-formylated proteins/peptides and small compounds^20,21^. Upon binding to formyl-peptide ligands, FPR1 elicits many responses including actin polarization, cell motility, production of reactive-oxygen species and release of cytokines by activating p38MAPK and PI3K/AKT signaling cascades^22,23^. Recently, FPR1 has been documented to be expressed also in non-myeloid cells, and many studies show that increased expression of FPR1 correlates with a poor prognosis in tumors of different origin^22,24–29^.

Some years ago, we generated libraries of uPAR-derived synthetic linear penta- and tetra-peptides, and peptidomimetic analogs carrying the Ser90 substituted with a glutamic acid residue or with the Cα-methyl-α-aminoisobutyric acid (Aib) and lead compounds were selected for their ability to inhibit uPAR/FPR1 interaction and reduce to basal levels cell migration and angiogenesis^15,30–32^. Although the proof-of-principle for this strategy was provided, none of these peptides revealed to be an ideal lead molecule since some of them are unstable to enzymatic digestion in human serum, which limits their half-life *in vivo*^15,30,31^, whereas the potent anti-angiogenic peptide Ac-L-Arg-Aib-L-Arg-D-Ca(Me)Phe-NH2 (UPARANT), which is stable in blood and reveals prolonged resistance to enzymatic proteolysis^32^, elicited some toxicity when systemically administered (unpublished).

To overcome these liabilities, we applied the Retro-Inverso (RI) approach to these uPAR/FPR1 inhibitors. The retro-inverso Ac-(D)-Tyr-(D)-Arg-Aib-(D)-Arg-NH2 (RI-3) peptide was selected as the best inhibitor of the uPAR-mediated FPR1-dependent cell migration^33^. RI-3 is stable in human serum, adopts the turned structure typical of uPAR/FPR1 antagonists and competes with fMLF and SRSRY for binding to FPR1. RI-3 prevents fMLF-induced FPR1 internalization as well as p38 MAPK and PI3K/AKT signaling cascades, which are documented to mediate fMLF-triggered signal transduction pathways^23,33–35^. Furthermore, in the absence of any stimulus, RI-3 did not change the phosphorylation of p38 MAPK and PI3K/AKT protein kinases^33^.

The pattern of activities of RI-3 prompted us to investigate the structure-function relationship between FPR1 and the fMLF, SRSRY and RI-3 peptides. This paper was organized according to the following objectives. The first one implied the molecular modeling of FPR1. The second objective was to study the structure-flexibility-function relationships of FPR1/fMLF and FPR1/SRSRY complexes by Molecular Dynamics (MD) and molecular docking simulations. Finally, we attempted to model and analyze FPR1/RI-3 complex in order to correlate RI-3 biological activity with the network of interaction between amino acids that signal to FPR1 C-terminal loop upon RI-3 engagement.

## Results

### FPR1 model

The 3D structure of the N-terminal and trans-membrane regions of FPR1 was modeled using comparative modeling and, as template, the structure of type-1 angiotensin II receptor (AGTR1, PDB code: 4YAY, UniProt code: P30556) because it showed the highest sequence identity (32%) with the FPR1 sequence by Blast search^36^. In the Supplementary Fig. S1 we report the alignment between the FPR1 and AGTR1 sequences. The best obtained model had a Prosa Z score of −2.51 and 97.7% of residues in most favored regions in the Ramachandran Plot.

**Figure 1:**
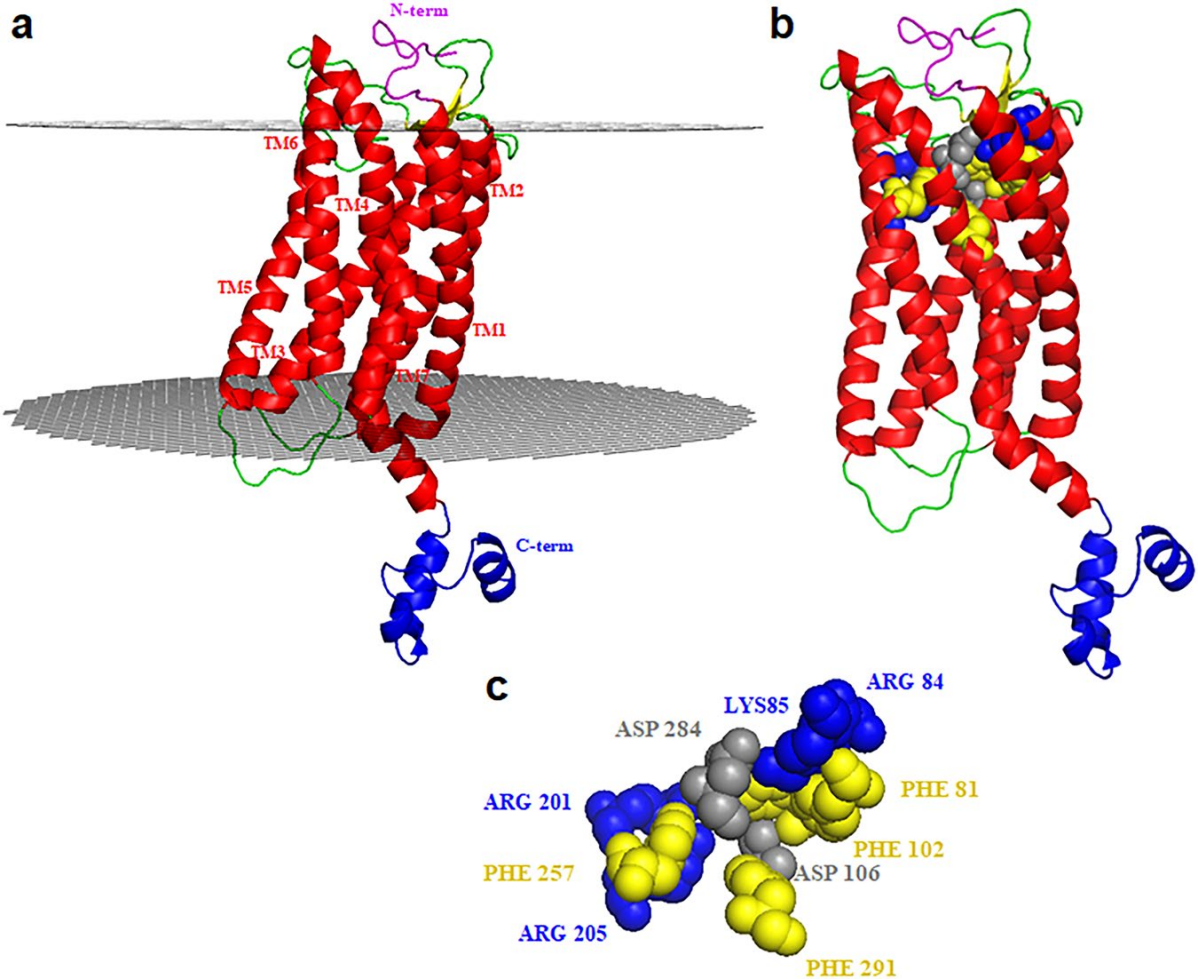
The 3D model of human FPR1. (**a**) The N-terminal region is reported in magenta whereas the C-terminal region in blue. For the trans-membrane region, we report the helices in red, the membrane boundaries in grey and the loop segments in green. (**b**) FPR1 with the binding pocket in which positive and negative charged residues are reported in blue and grey, respectively, whereas aromatic residues are reported in yellow. (**c**) Snapshot of the binding pocket, using the same orientation of (**b**), in which positive and negative charged residues are reported in blue and grey, respectively, whereas aromatic residues are reported in yellow.

Then, since in the case of C-terminal domain of FPR1 we did not find structures that can be used as templates, we modelled this domain using *ab initio* approach of Quark server^37^, and selected the best model on the basis of TM-score, and energetic and stereo-chemical quality as described in our recent paper^38^. The best elected model had a TM-score equal to 0.37, an energetic Z-score of −4.28, and 94.3% of the residues in the allowed regions in the Ramachandran Plot. Finally, the complete structural model of FPR1 was obtained by comparative modeling, using as templates the obtained models for N-terminal and trans-membrane regions and for C-terminal domain. The final structure had 96.8% of the residues in allowed regions in the Ramachandran Plot, and an energetic score of −3.28. As shown in Fig. 1a, the whole model of FPR1 comprised: i) a disordered N-terminal segment; ii) seven trans-membrane helices (TM1-TM7) with six loops among which three in the extracellular region and other three in the cytoplasm region; iii) a C-terminal region located in the cytoplasm composed by three short helices. In particular, the extracellular loop 2 comprises two short β-strands in agreement with other trans-membrane receptors^39^. FPR1 presents a well-defined binding pocket characterized by positively charged residues like Arg84 and Lys85 located in TM2 and Arg201 and Arg205 on TM5, and by the negatively charged residues Asp284 on TM7and Asp106 on TM3. However, between these two zones there are also hydrophobic residues that separate these charged areas and interact with the ligands. In detail, from one side there are Phe81 on TM2 and Phe102 on TM3 that form the anchor region with Arg84 and Lys85 located always on TM2 whereas from the other side there are Tyr257 on TM6 and Phe291 on TM7 that form the activation region with Arg201 and Arg205 located on TM5 (Fig. 1b,c). This is in agreement with the known ability of FPR1 to recognize a variety of ligands with different chemical properties and origins^20^. It is important to highlight that: i) the binding site, that is “cone-shaped with the tip towards the trans-membrane region”, is small and can accommodate only one peptide at a time considering that its radius (of about 8 Å) and its height (of about 13 Å) are lower than the length of the peptides (of about 14 Å); ii) the charged and aromatic residues in the pocket allow the charged and aromatic residues of the peptides to strongly interact with FPR1 by ionic-aromatic and stacking interactions, H-bonds, and salt bridges.

### Complex between fMLF and FPR1

To characterize the interactions between FPR1 and fMLF, we first performed MD simulations on fMLF, linearly modeled as reported in the Methods. In Supplementary Fig. S2a, it is shown the Root Mean Square Deviation (RMSD) plot computed by overlapping the various structures during the simulations with all the atoms compared to initial conformation. RMSD plot of fMLF shows high levels of fluctuation with RMSD values ranging between 0.02 and 0.08 nm, which suggest that this peptide is flexible. This finding was further confirmed by Root Mean Square Fluctuations (RMSF) plot where the most flexible residue during the simulation is the phenylalanine (Supplementary Fig. S2b) with RMSF value equal to 0.25 nm. Also, the radii gyration (RG) plot (Supplementary Fig. S2c) show that the peptide is flexible; in fact, RG values range between 0.25 and 0.29 nm during MD. The analysis of H-bonds at 0, 20, 40, 60, 80 and 100 ns did not show the presence of main chain-main chain (MM) H-bonds suggesting that the conformation of fMLF is extended. This final conformation obtained for fMLF after MD (Supplementary Fig. S2d) was used for further docking studies modeling its interaction with FPR1.

**Figure 2:**
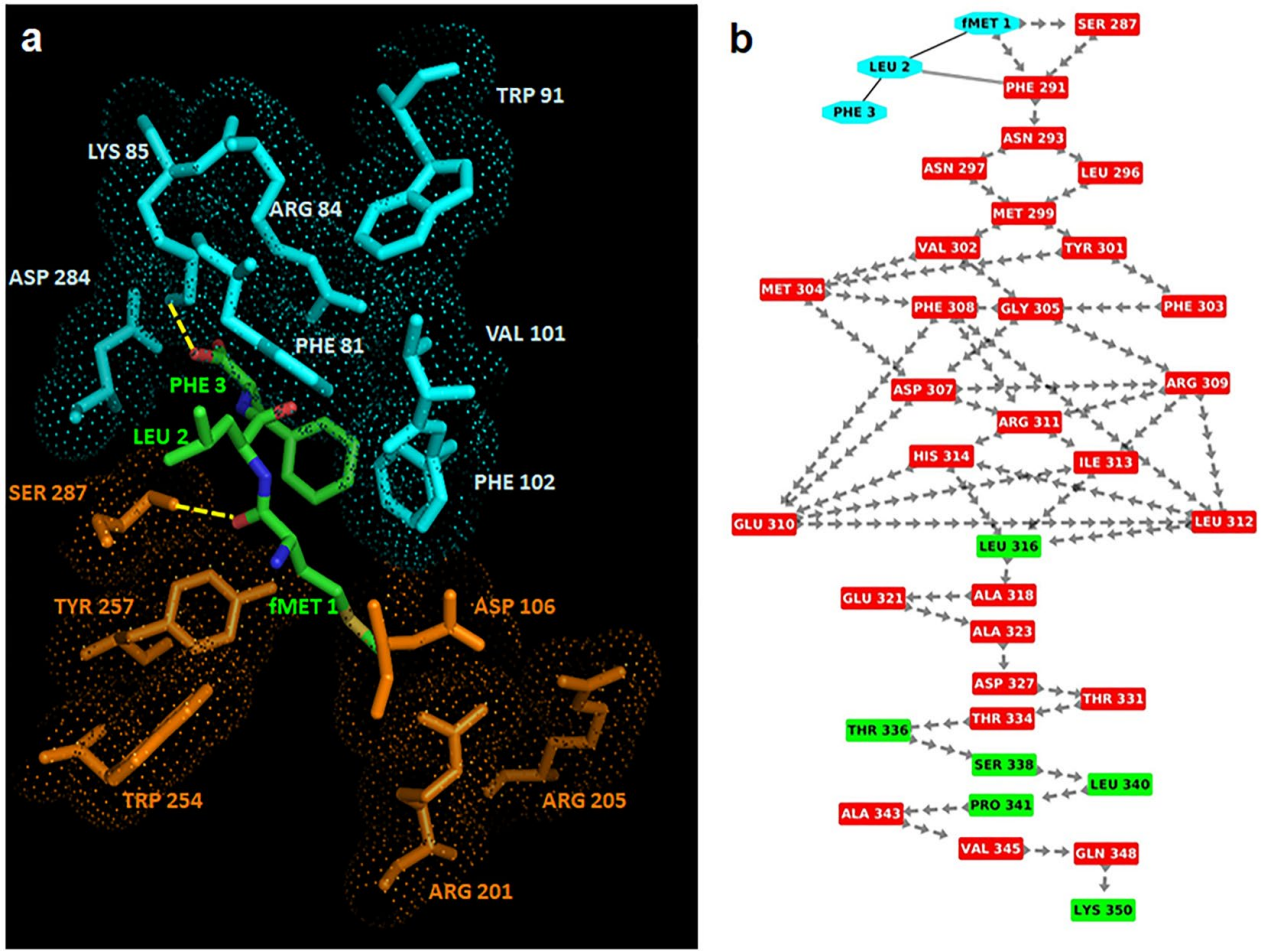
Interactions between fMLF and FPR1. (**a**) The residues in the activation and anchor region of FPR1 are depicted in orange and cyan, respectively, whereas the residues of fMLF in green. H-bonds are highlighted in yellow. (**b**) Snapshot of interaction network (the “activation signature”) going from fMLF to the C-terminal loop of FPR1 through crossing of helix 7. Residues of the fMLF peptide are shown in cyan whereas residues in α-helix and loop in red and green, respectively. The peptide bonds are shown as black lines whereas the interactions with their closest atoms (IAC) and H-bonds by grey double lines and arrows, respectively.

The best scored binding configuration between fMLF and FPR1 was selected on the basis of the number of interacting residues, H-bonds and salt bridges. In this complex, Phe3 is surrounded by: i) Phe81, Arg84 and Lys85 on TM2 that are reported as anchor region; ii) Trp91 on extracellular loop 1; iii) Val101 and Phe102 on TM3; iv) Asp284 on TM7. Hence, the interaction of Phe3 with FPR1 is based on three charged and four hydrophobic residues (Fig. 2a,b). On the other hand, the formylated Met1 is surrounded by: i) Asp106 on TM3; ii) Arg201 and Arg205 on TM5 (specific for activation region); iii) Trp254 and Tyr257 (specific for activation region) on TM6; iv) Ser287 on TM7. However, it is important to underline that the formylated Met1 binds the activation region and this interaction is stabilized by a MM H-bond with Ser287 on TM7, whereas Phe3 interacts with the anchor region through a stacking bond with Phe102 and a MM H-bond with Lys85.

**Figure 3:**
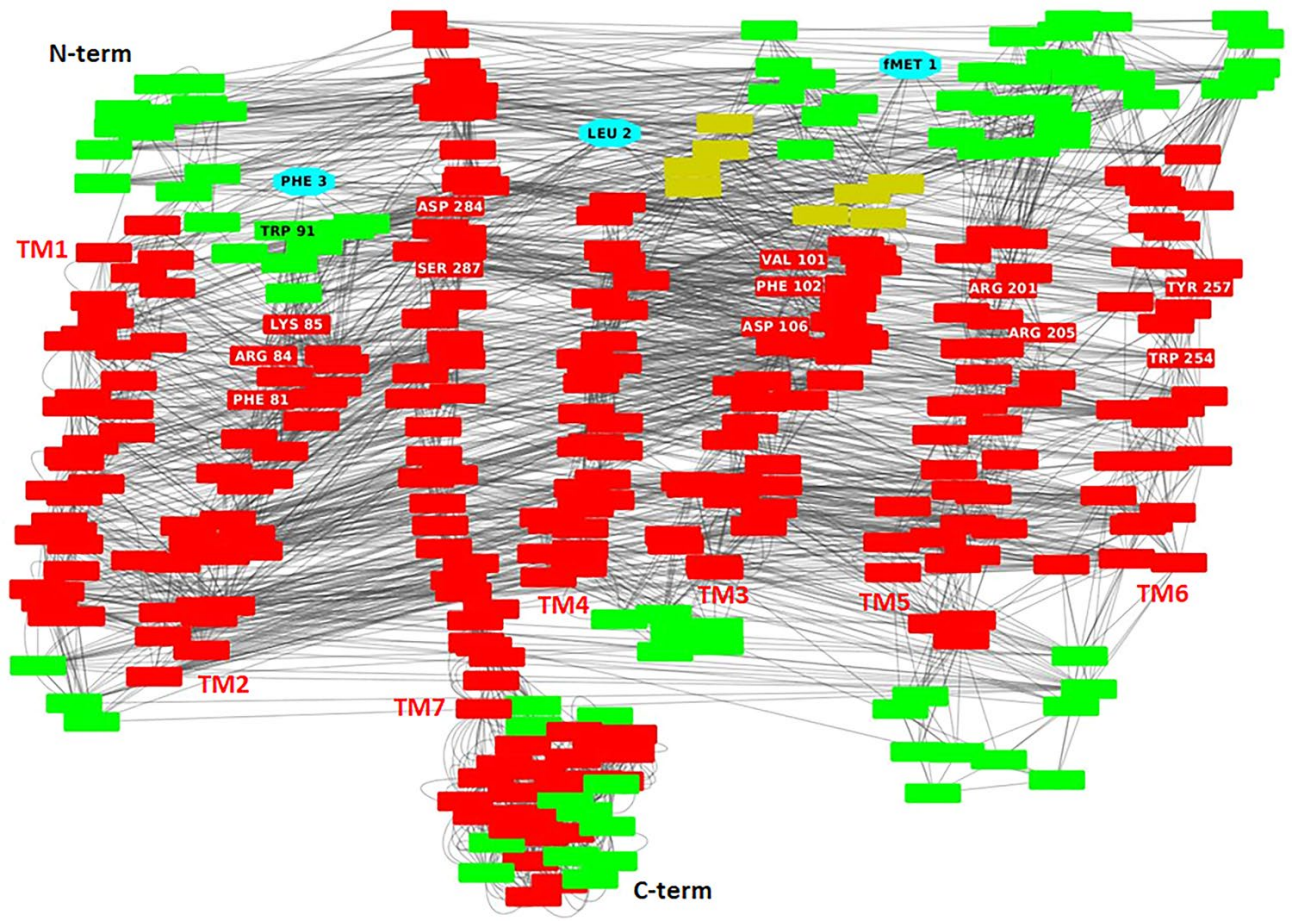
Interaction network of the complex between fMLF and FPR1. Residue interaction network related to the complex between fMLF and FPR1 where the nodes (reported by red rectangular boxes) are the amino acids whereas the edges are the interactions between the residues (like H-bonds, π-cations, π-stacking and interactions with their closest atoms (IAC). In details, the residues of the fMLF peptide are shown by cyan nodes whereas the residues of FPR1 in helices, loops and β-strands by red, green and yellow nodes, respectively. Labels show the localization of the seven helices, the N-terminal and C-terminal regions. Moreover, the residues present in the activation and anchor regions of FPR1 are evidenced by white labels in red nodes if located in helices and by black labels in green nodes if located in loops.

To understand how fMLF is able to activate FPR1 and to function as an agonist, we created an interaction network between amino acids, based on the interaction structure of residues (Fig. 3). This approach allows to identify those residues which have the strongest role in the signaling^38^. The analysis uncovered the occurrence of a network of MM H-bonds that starts from the H-bonds between the formylated Met1 of fMLF and Ser287, and proceeds from Ser287 to the C-terminal region by crossing TM7 (Fig. 2b). This network of interactions transmits the conformational transition following agonist binding that results in the initiation of the signaling cascade. We see it as signature (an “activation signature”) that defines fMLF as an agonist. Taken together, our findings indicate that: i) fMLF is flexible and recognizes FPR1 in a preferentially extended conformation, as already reported by He and coworkers^40^; ii) the binding pocket of FPR1 comprises a localized region between the transmembrane helices TM2, TM3, TM5, TM6 and TM7; and iii) fMLF-engaged FPR1 displays an activation signature which reaches the FPR1 C-terminal loop by crossing helix 7.

It is documented that the C-terminal region is crucial for internalization, desensitization, and arrestin2 binding and that Ser328, Ser332, and Ser338 located in this region regulate these processes^41^. In our network, Ser328 H-bonds with Leu324 and Ser332, and in turn it forms H-bond with Gln330, whereas Ser338 forms two H-bonds with Thr336 and Leu340. These findings confirm the central role of these three residues in the H-bond network involved in signaling, and fit well with the ability of fMLF to trigger FPR1 internalization and signaling upon binding to FPR1^15^.

### Complex between SRSRY and FPR1

To characterize the interactions between FPR1 and SRSRY, MD simulations were performed on the SRSRY peptide, linearly modeled as reported in the Methods. RMSD plot for this peptide shows fluctuations with the highest RMSD values equal to 0.35 nm after 20 ns and 60 ns (Supplementary Fig. S3a). RMSF plot evidences that the most flexible residues are Ser1 and Tyr5 (Supplementary Fig. S3b). On the other hand, Supplementary Fig. S3c shows a decrease of RG values at 20 and 60 ns reaching an RG value of 0.33 nm, suggesting that the peptide tends to become more compact and, thus, the radius of gyration decreases. This is also confirmed by the presence of one MM H-bond between the CO group of Ser1 and NH group of Arg4 at 20 ns and two MM H-bonds between the CO group of Ser1 and the NH group of Arg4 and between the CO group of Ser1 and the NH group of Tyr5, suggesting that SRSRY tends to form a turn (Supplementary Fig. S3d).

Since during MD this peptide alternates extended and turn conformations as previously shown^30^, we used both conformations in further docking studies with FPR1.

**Figure 4:**
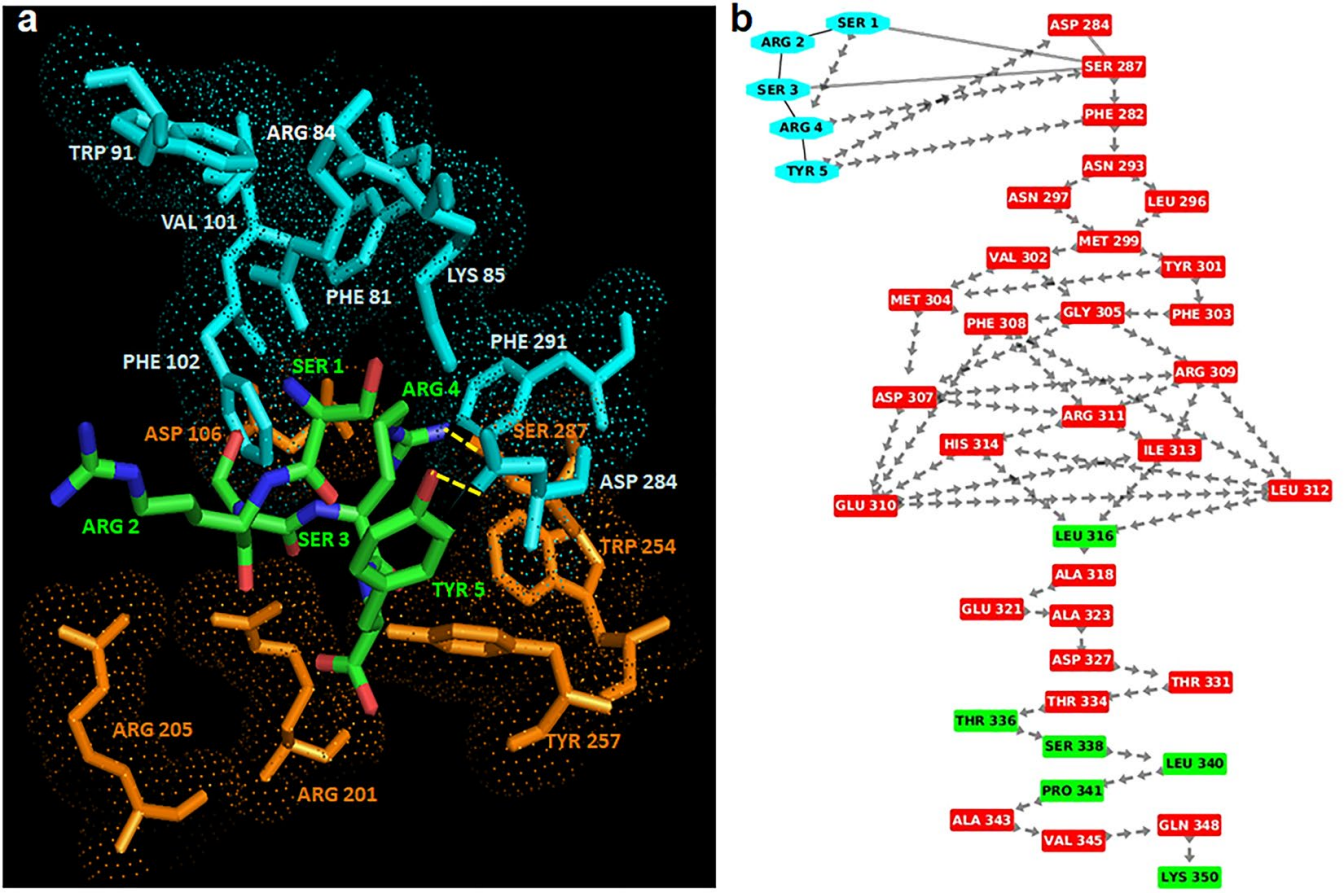
Interaction between FPR1 and SRSRY in turn conformation. (**a**) The residues in the activation and anchor region of FPR1 are depicted in orange and cyan, respectively, whereas the residues of SRSRY in turn in green. H-bonds are shown in yellow. (**b**) Interaction network (the “activation signature”) going from SRSRY(turn) to the C-terminal loop of FPR1 through crossing of helix 7. Residues of SRSRY(turn) are shown in cyan whereas residues in α-helix and loop are depicted in red and green, respectively. The peptide bonds are shown as black lines whereas the interactions with their closest atoms (IAC) and H-bonds by grey double lines and arrows, respectively.

In the case of SRSRY in turn conformation, SRSRY (turn), the best scored binding configuration between the peptide and FPR1 was selected on the basis of the number of interacting residues, H-bonds and salt bridges. In this complex (Fig. 4a), Tyr5 is surrounded by Phe81, Arg84 and Lys85 on TM2, Trp91 on extracellular loop 1, Val101 and Phe102 on TM3, Asp284 and Phe291 on TM7, whereas Arg4 is surrounded by Asp106 on TM3, Arg201 and Arg205 on TM5, Trp254 and Tyr257 on TM6 and Ser287 on TM7. In detail, Tyr5 forms a H-bond with Asp284 located on TM2 in the anchor region and Arg4 forms a MM H-bond with Ser287.

Comparing the two complexes fMLF/FPR1 and SRSRY(turn)/FPR1, we found that in the SRSRY(turn)/FPR1 complex: i) there is also Phe291 of FPR1 in the anchor region; ii) the interaction between SRSRY(turn) and FPR1 in the anchor region is stabilized not only by the H-bond between Tyr5 and Lys85, but also by an additional H-bond between Tyr5 and Asp284. These observations suggest that the binding sites of fMLF and SRSRY (turn) are very similar/adjacent, even if not perfectly identical.

The analysis based on interaction network of the complex between SRSRY(turn) and FPR1 highlighted that, as for the fMLF-FPR1 complex, there is a network of MM H-bonds that starts from the H-bonds between Arg4 of SRSRY and Ser287 and proceeds from Ser287 to the C-terminal region by crossing TM7 (Fig. 4b). This suggests the activation signature of SRSRY(turn) is the same found in fMLF/FPR1 complex.

**Figure 5:**
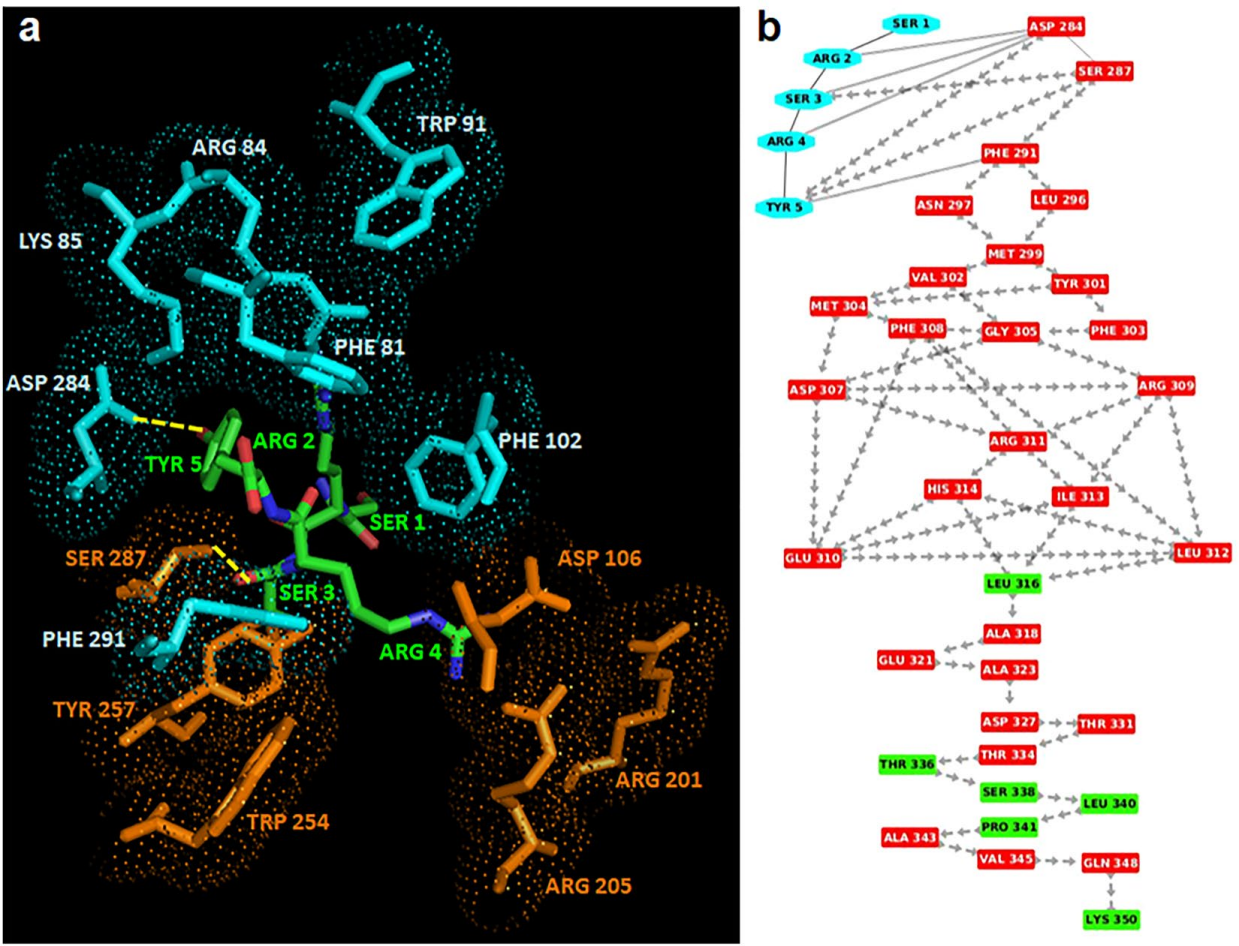
Interactions between FPR1 and SRSRY in extended conformation. (**a**) The residues in the activation and anchor region of FPR1 are depicted in orange and cyan, respectively, whereas the residues of extended SRSRY in green. H-bonds are shown in yellow. (**b**) Interaction network (the “activation signature”) going from SRSRY (extended) to the C-terminal loop of FPR1 through crossing of helix 7. Residues of SRSRY (extended) are shown in cyan whereas residues in α-helix and loop are depicted in red and green, respectively. The peptide bonds are shown as black lines whereas the interactions with their closest atoms (IAC) and H-bonds by grey double lines and arrows, respectively.

Then, we modeled the complex between the extended conformation of SRSRY, SRSRY(extended), and FPR1. In this complex, Tyr5 is surrounded by Phe81, Arg84 and Lys85 on TM2, Trp91 and Phe93 on extracellular loop1, Asp284 and Phe291 on TM7, whereas Ser3 is in contact with Asp106 on TM3, Arg201 and Arg205 on TM5, Trp254 and Trp257 on TM6 and Ser287 on TM7 (Fig. 5a). In the extended conformation of SRSRY, Tyr5 is in contact with Phe93, a contact that was absent in the fMLF/FPR1 and SRSRY(turn)/FPR1complexes, while the interactions with Val101 and Phe102 located on TM3 are lost. Hence, in the extended conformation SRSRY continues to interact with FPR1, but the binding site is not identical and is less stabilized with respect to the fMLF/FPR1 and SRSRY turn)/FPR1 complexes. In SRSRY(extended), Tyr5 forms a H-bond with Asp284 and a π-cation interaction with Lys85, suggesting that SRSRY(extended) interacts as strongly with the anchor region as in the complex between FPR1 and SRSRY(turn). Regarding the activation region, we find that Ser3 of SRSRY(extended) forms a main chain-side chain (MS) H-bond with Ser287 of FPR1, suggesting that this peptide can still activate the signaling pathway, but weaker than in the turn conformation (Fig. 5b). Collectively, these findings strongly support the notion that fMLF and SRSRY use the same activation signature, with a very similar/adjacent binding site on FPR1, located between TM2, TM3, TM5, TM6 and TM7.

**Figure 6:**
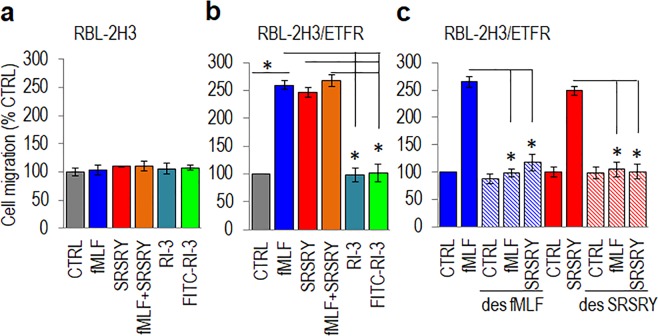
Effect of FPR1 peptide agonists and antagonists on cell migration. (**a**,**b**) RBL-2H3 (**a**) and RBL-2H3/ETFR (**b**) cells were allowed to migrate toward serum-free medium (CTRL), the indicated peptides at 10 nM concentration or 10 nM fMLF mixed to equimolar concentration of SRSRY in Boyden chambers for 4 h. (**c**) RBL-2H3/ETFR cells were exposed to diluents or desensitized with 100 nM fMLF or 100 nM SRSRY for 30 min at 37 °C and then allowed to migrate in Boyden chambers for 4 h at 37 °C toward serum-free medium (CTRL), 10 nM fMLF or 10 nM SRSRY. In all cases the extent of migration is expressed as a percentage of the basal cell migration considered as 100% (CTRL) and all values are reported relative to that. Data are the means ± SD of three independent experiments, performed in triplicate. Statistical significance with *p < *0*.*001*.

Accordingly, we found that, like fMLF, 10 nM SRSRY promotes directional migration of only RBL-2H3 cells stably transfected with human FPR1 (RBL-2H3/ETFR), the extent of fMLF and SRSRY cell motility being quite comparable (Fig. 6a,b). Interestingly, when cell migration was directed toward a mix of 10 nM fMLF and 10 nM SRSRY, the extent of directional RBL-2H3/ETFR cell migration did not change significantly (Fig. 6b). This, combined with the finding that cells desensitized with fMLF or SRSRY fail to migrate to SRSRY or fMLF, respectively, (Fig. 6c), definitely indicate that fMLF and SRSRY share an adjacent binding site on FPR1 and signal through the same conformational transition.

### Complex between RI-3 and FPR1

**Figure 7:**
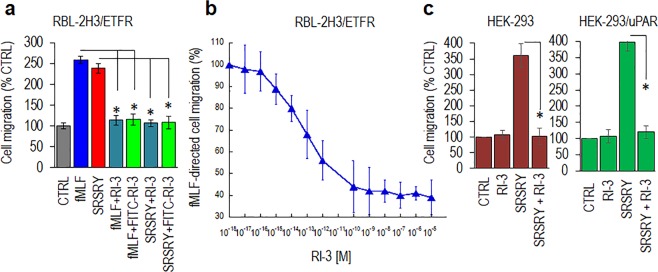
RI-3 inhibits migration of FPR1 expressing cells in a dose dependent manner. (**a**) RBL-2H3/ETFR cells were allowed to migrate toward serum-free medium (CTRL) or the indicated peptides at 10 nM concentration in Boyden chambers for 4 h. The extent of migration is expressed as a percentage of the basal cell migration considered as 100% (CTRL) and all values are reported relative to that. (**b**) RBL-2H3/ETFR cells were allowed to migrate toward 10 nM fMLF mixed to increasing concentration of RI-3 in Boyden chambers for 4 h. The extent of migration is expressed as a percentage of cell migration toward fMLF alone, considered as 100%, and all values are reported relative to that. (**c**) HEK-293 and HEK-293/uPAR cells were allowed to migrate toward serum-free medium (CTRL) or the indicated peptides at 10 nM concentration in Boyden chambers for 4 h. The extent of migration is expressed as a percentage of the basal cell migration considered as 100% (CTRL) and all values are reported relative to that. In all cases, data are the means ± SD of three independent experiments, performed in triplicate. Statistical significance with *p < *0*.*001*.

In a recent paper we showed that RI-3 prevents fMLF-induced FPR1 internalization and inhibits sarcoma cell migration in a dose dependent manner^33^. Now, we present evidence that RI-3 failed to trigger migration of both FPR1-lacking and FPR1-expressing RBL-2H3 cells (Fig. 6a,b) but reduced to the basal levels the migration of RBL‐2H3/ETFR cells directed not only toward 10 nM fMLF, but also toward 10 nM SRSRY (Fig. 7a). Inhibition of cell migration by RI-3 is dose-dependent as it starts in the high fM range, it seems to level off in the low nM range, an overall 50% reduction of cell migration being reached at 1 × 10^−13^ M (Fig. 7b). Similar results were obtained when human HEK-293 cells which do not express uPAR and HEK-293 cells stably transfected with uPAR cDNA, were allowed to migrate toward 10 nM SRSRY. According to previously reported data^13^, SRSRY peptide elicited an appreciable cell migration of either HEK-293 and HEK-293/uPAR cells. In both cases, SRSRY-directed cell migration was reduced to the basal level by 10 nM RI-3 (Fig. 7c).

**Figure 8:**
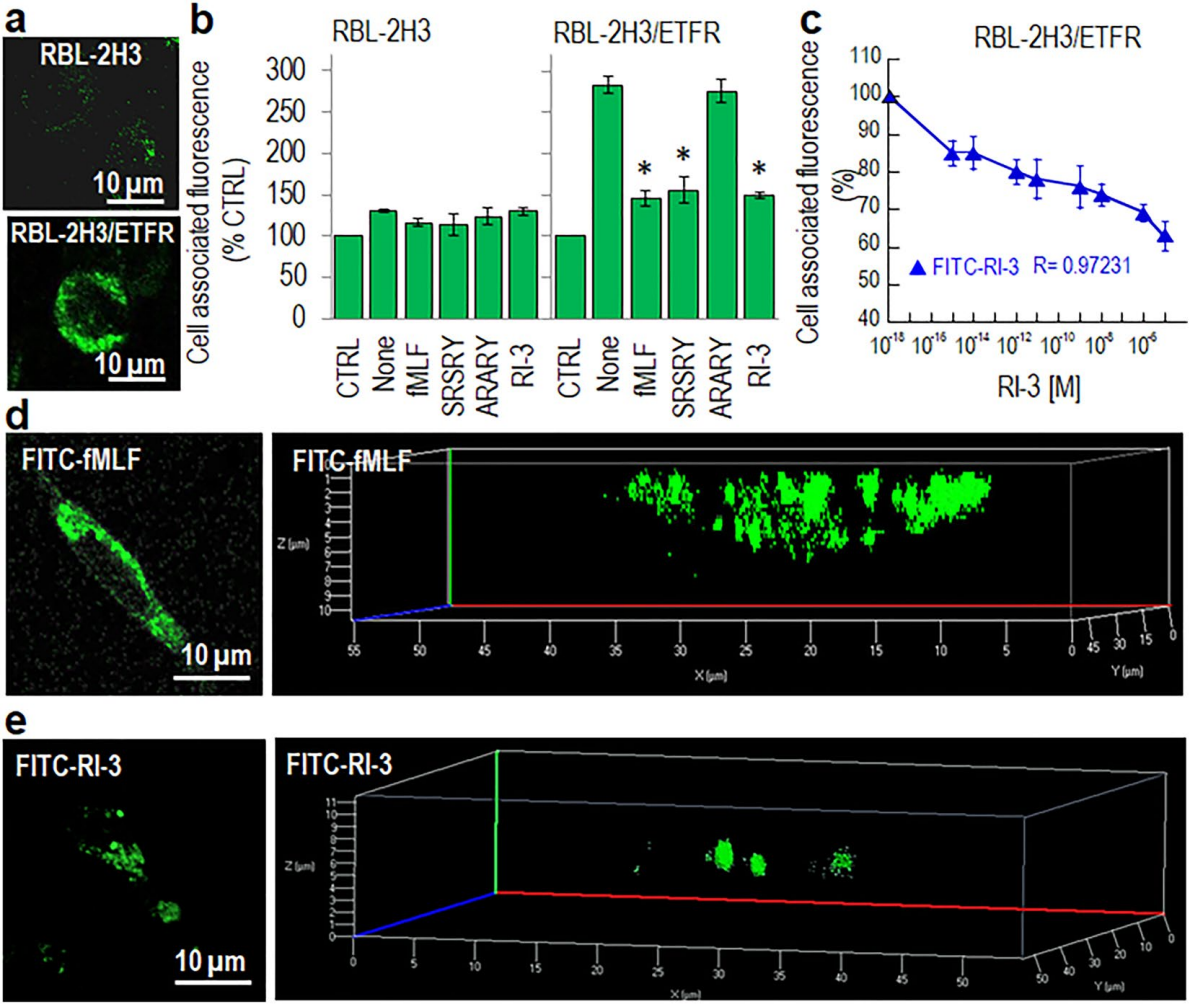
Binding properties of RI-3. (**a**) Images of RBL-2H3 and RBL-2H3/ETFR cells exposed to 10 nM FITC-RI-3 for 45 min at 4 °C and then visualized using a Zeiss 510 Meta LSM microscope. Original magnification: 630x. (**b**) RBL-2H3 and RBL-2H3/ETFR cells (1.5 × 10^6^ cells/sample) were pre-incubated with diluents (None), 1 µM fMLF, 1 µM SRSRY, 1 µM ARARY, or 1 µM RI-3 for 30 min at 4 °C and then exposed to 10 nM FITC-RI-3 for additional 45 min at 4 °C. Fluorometric measurement of cell-associated fluorescence was assed using 485 nm excitation and 535 nm emission filters. Data are expressed as a percentage of the basal fluorescence intensity, normalized to 100% (CTRL) and represent a mean ± SD from three independent experiments, performed in triplicate. *Statistical significance against None with *p < *0*.*01*. (**c**) Fluorometric measurement of cell-associated fluorescence of RBL-2H3/ETFR cells incubated with increasing i concentrations of RI-3 for 30 min at 4 °C and then exposed to 10 nM FITC-RI-3 for additional 45 min at 4 °C. Data are expressed as a percentage of surface-associated fluorescence assessed in the absence of RI-3, normalized to 100% and represent a mean ± SD from three independent experiments, performed in triplicate. (**d**,**e**) Images of RBL-2H3/ETFR cells grown adherent on glass slides to semi-confluence, exposed to 10 nM FITC-fMLF (**d**) or 10 nM FITC-RI-3 (**e**) for 30 min at 37 °C and then visualized using a Zeiss 510 Meta LSM microscope in 2D (left) and 3D (right) projections. Original magnification: 630x.

To investigate whether RI-3 directly binds to FPR1, we took advantage of a fluorescein-conjugated RI-3 peptide (FITC-RI-3) which does not exert any chemotactic activity and inhibits both fMLF- and SRSRY-directed cell migration to a similar extent as compared to unlabeled RI-3 (Figs 6a,b and 7a). Competition binding assays were carried out on RBL-2H3 and RBL-2H3/ETFR cells exposed to 10 nM FITC-RI-3 in the presence of an excess of fMLF, SRSRY, RI-3, a control inactive peptide ARARY^13^, or increasing concentration of RI-3 at 4 °C, to avoid any FPR1 internalization. FITC-RI-3 uptake was observed on the surface of RBL-2H3/ETFR but not RBL-2H3 cells (Fig. 8a). Fluorometric measurement of cell associated fluorescence confirmed the absence of any specific FITC-RI-3 uptake on RBL-2H3 cells (Fig. 8b). In contrast, we found a specific binding of FITC-RI-3 to RBL-2H3/ETFR cell surface that was abrogated by pre-incubation with an excess of fMLF, SRSRY or RI-3, while ARARY was ineffective (Fig. 8b). FITC-RI-3 uptake on RBL-2H3/ETFR cells starts in the fM range, 50% inhibition being observed at 1 × 10^−11^ M RI-3 (Fig. 8c).

To assess whether RI-3 itself causes FPR1 internalization, RBL-2H3/ETFR, grown adherent on glass slides, were exposed to 10 nM FITC-fMLF or 10 nM FITC-RI-3 for 30 min at 37 °C and then visualized with a confocal microscope. As expected, FPR1 appeared mainly internalized in RBL-2H3/ETFR cells exposed to FITC-fMLF as indicated by punctuate green fluorescent intra-cytoplasmic spots (Fig. 8d) which were only slightly detectable in cells exposed toFITC-RI-3 (Fig. 8e). Z-stack analysis of the images recorded with 0.19 µm intervals through the entire cell thickness and visualized in 3D projection. confirmed the paucity of RI-3 internalization as compared with RBL-2H3/ETFR cells exposed to FITC-fMLF (Fig. 8d,e, Supplementary Figs S4 and S5), suggesting a mechanism in which, in the absence of agonist, RI-3 binds to FPR1 keeping it anchored to the membrane and unable to signal. Furthermore, the higher binding affinity of RI-3 for FPR1, as compared to fMLF and SRSRY, whose binding affinities are in the nanomolar range^13,42^, ensures its capability to prevent engagement, internalization and signaling of FPR1 by either fMLF and SRSRY. Binding experiments carried out on HEK-293 and HEK-293/uPAR cells that are documented to express FPR1^43^, allowed us to ascertain that, when expressed on cell surface, uPAR does not affect the binding of FITC-RI-3 to FPR1. Moreover, unlike FITC-fMLF, FITC-RI-3 does not trigger FPR1 internalization in HEK-293/uPAR cells (Supplementary Fig. S6).

Previously performed MD simulations on RI-3 peptide had shown that: i) RI-3 reached convergence after 20 ns with a mean value of RMSD around 0.3 nm; ii) RG plot for this peptide tended to decrease; iii) RI-3 formed a turn structure during the great part of the MD simulation with the formation of one MM H-bond between the CO group of D-Tyr1 and NH group of D-Arg4, specific of turn conformation^33^. Therefore, the final conformation in which RI-3 forms a turn was used in further docking studies with FPR1.

**Figure 9:**
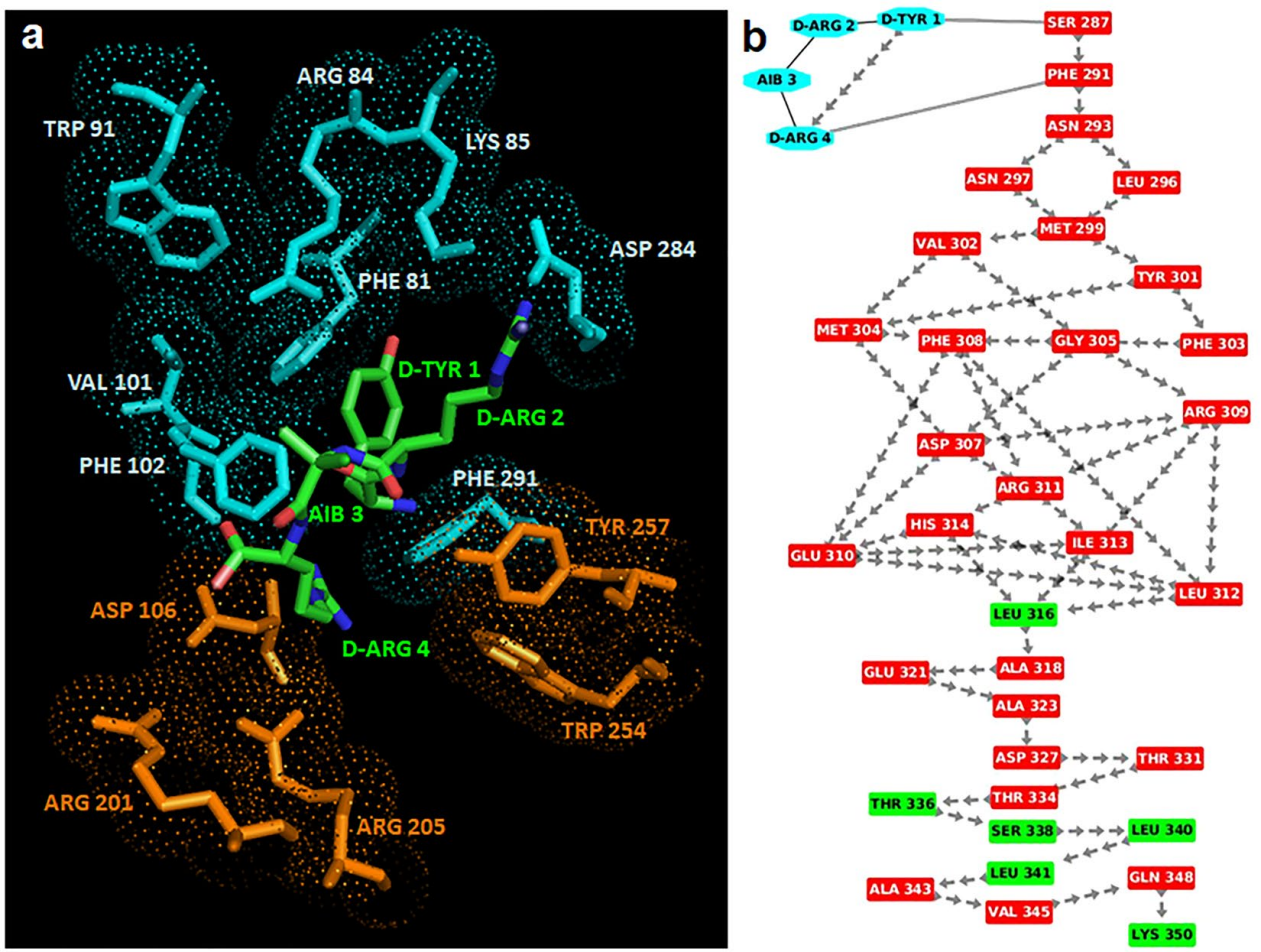
Interaction between RI-3 and FPR1. (**a**) The residues in the activation and anchor region of FPR1 are depicted in orange and cyan, respectively, whereas the residues of RI-3 in turn conformation are shown in green. H-bonds are shown in yellow. (**b**) Snapshot of interaction network from RI-3 to the C-terminal loop of FPR1 through crossing of helix 7. Residues of RI-3 are shown in cyan whereas residues in α-helix and loop are depicted in red and green respectively. The peptide bonds are shown as black lines whereas the interactions with their closest atoms (IAC) and H-bonds by grey double line and arrows, respectively.

The best scored binding configuration between RI-3 and FPR1 was selected on the basis of the number of interacting residues, H-bonds and salt bridges. In this complex, D-Tyr1 is surrounded by Phe81, Arg84 and Lys85 on TM2, Trp91 on the extracellular loop1, Val101 and Phe102 on TM3 and Asp284 and Phe291 on TM7 whereas the residues Aib3 and D-Arg4 are surrounded by Asp106 on TM3, Arg201 and Arg205 on TM5 and Trp254 and Tyr257 on TM6 (Fig. 9a). In detail, D-Tyr1 interacts with Phe81, Phe102 and Phe291 by stacking and with Arg84 by π-cation interaction, indicating that RI-3 strongly interacts with the anchor region. On the other hand, D-Arg4 does not form H-bonds with the residues in the activation region and on TM7, indicating that the signaling is not activated (Fig. 9b).

Altogether, our biological findings fit well with MD simulations of the FPR1/RI-3 complex. RI-3 competes with fMLF and SRSRY for binding to FPR1 and inhibits both fMLF- and SRSRY-directed cell migration by preventing agonist-triggered FPR1 internalization. Accordingly, RI-3 binds strongly FPR1 in the anchor region and shares the same binding site of fMLF and SRSRY on FPR1, located between TM2, TM3, TM5, TM6 and TM7 helices, but fails to engage the residues in the activation region. Therefore, in the absence of any stimulus, RI-3 does not promote neither cell migration, nor FPR1 internalization.

## Discussion

Cell migration offers rich targets for intervention since its control may improve pathologic conditions sustained by an altered cell motility, including chronic inflammation and neoplastic diseases.

In the past years, we developed uPAR-derived synthetic peptides that inhibit the uPAR/FPR1 interaction and reduce to the basal level the motility of cancer cells^15,30–32^. More recently, in order to overcome the liabilities of these inhibitors due to their peptidic nature, we developed new retro-inverso peptides. Among these, the retro-inverso peptide RI-3 was identified as the best inhibitor of cell migration. RI-3 adopts a turn structure very similar to that adopted by previous characterized uPAR-FPR1 antagonists, is stable in human serum, prevents the uPAR/FPR1 interaction and potently inhibits migration and invasion of human sarcoma and melanoma cells^15,30,31,33,34^. Furthermore, RI-3 does not change the phosphorylation of protein kinases in the absence of any stimulus, but prevents fMLF-triggered activation of p38 MAPK and PI3K/AKT signaling cascades, without affecting cell proliferation^33,34^.

To understand the structural basis of RI-3 inhibitory effect on cell migration, in the absence of published crystallographic structures of FPR1, we decided to model the structure of this receptor and of the three FPR1/fMLF, FPR1/SRSRY and FPR1/RI-3 complexes, focusing the attention to the binding pocket of FPR1 and the interaction network of amino acids that transmits the binding information throughout the complex to the FPR1 C-terminal loop, triggering the signaling cascade.

In keeping with the notion that the ligand binding pocket of FPR1 consists of several key residues located in different transmembrane helices^21,44,45^, we identified a well-defined binding pocket of FPR1 which can accommodate only one peptide at a time, and is characterized by positively charged residues located in TM2 and TM5, by negatively charged residues on TM3, and by aromatic residues on TM6 and TM7. The peptides interact strongly with the charged and aromatic residues in the pocket by H-bonds, salt bridges and ionic-aromatic and stacking interactions. Comparison of the FPR1/fMLF and FPR1/SRSRY complexes uncovered that fMLF and SRSRY share a similar/adjacent binding site on FPR1, located between TM2, TM3, TM5, TM6 and TM7, and that the network of interactions through which SRSRY triggers signaling is the same found in the fMLF/FPR1 complex. fMLF recognizes FPR1 in a preferentially extended conformation as already reported by He and coworkers^40^, whereas SRSRY is more flexible and recognizes FPR1 either in extended or in compact conformation. However, in the extended conformation the binding of SRSRY to FPR1 is less stabilized than in the turn conformation. These differences may be explained considering that Ser90 is positioned in a critical hinge which influences the conformation of nearest residues^16^. Considering that uPAR may reversibly acquire distinct conformational states transitioning from an open and inactive to a closed and active conformation, and that uPA engagement shifts the inactive structure of uPAR to a closed, active conformation^11^, it is conceivable to hypothesize that FPR1/SRSRY(turn) and FPR1/SRSRY(extended) complexes could reflect intermediate stages of transition between the open/inactive to closed/active conformation of uPAR. Thus, uPA, beside focusing of its proteolytic activity on cell surface, could ultimately regulate the formation of uPAR/FPR1 complexes.

We also present evidence that RI-3 inhibits cell migration of human cells expressing a considerable amount of uPAR on the cell surface, in spite of the fact that RI-3 competes with SRSRY for binding to FPR1. This apparent contradiction may be reconciled considering that FPR1 displays an about 1000-fold higher binding affinity to RI-3 than to SRSRY (Kd:1 × 10^−11^ M, and 3 × 10^−8^ M, respectively^15^). The relevance of this observation is not obvious considering that uPAR overexpression has been found in tumor tissues of different origin and soluble forms of uPAR containing the uPAR84-95 sequence shed from tumor cells and enrich cancer microenvironment with a strong pro-chemotactic factor^6,46^. Furthermore, the finding that, unlike fMLF, RI-3 does not trigger FPR1 internalization, either in the presence, or in the absence of uPAR, suggests that FPR1 is necessary to promote cell migration, and that the potency of chemotactic uPAR84-95 sequence is mainly mediated by FPR1.

Previous work from this laboratory has shown that RI-3 forms a turn structure during the great part of the MD simulation, with the formation of one MM H-bond between the CO group of D-Tyr1 and NH group of D-Arg4, specific of a turn conformation^33^. The detailed analysis of the FPR1/RI-3 complex reported here shows that RI-3: i) shares the same binding site of fMLF and SRSRY on FPR1 located between TM2, TM3, TM5, TM6 and TM7 helices; ii) strongly binds FPR1 in the anchor region, as indicated by three stacking and one π-cation interactions in which D-Tyr1 is involved, and iii) does not interact with the activation region of FPR1, thus being unable to activate the signaling cascade. These observations fit well with the finding that RI-3 binds to FPR1 with high affinity (apparent Kd:1 × 10^−11^ M) without triggering its internalization, nor cell motility. In agreement with docking studies, RI-3 can be regarded as a true FPR1 antagonist, since it does not elicit by itself the migration of either FPR1-lacking or FPR1-expressing cells, but reduces to basal level the migration of FPR1-expressing cells toward both SRSRY and fMLF, the latter with an IC_50_ of 1 × 10^−13^ M.

The addition of an ε-aminocaproic acid spacer to the C-terminal amidated group of RI-3 in FITC-RI-3 did not modify significantly its binding properties. This information, together with the docking analysis of the FPR1/RI-3 complex may provide sufficient information for the design of peptide derivatives labeled with therapeutic radioisotopes, to elicit additional cytotoxic activity against cancer cells. Furthermore, the new compounds could be radiolabeled for micro-PET imaging in order to monitor their distribution and pharmacokinetics simultaneously in animal models.

During the last twenty years, a very large number of natural and synthetic compounds that interact and/or interfere with FPR1-dependent pathways and several agonists and antagonists of FPR1-dependent functions have been described^35,47^. In this context, the selective inhibition of the uPAR/FPR1 interaction could facilitate the development of selective inhibitors of the processes sustained by a chronic excess of cell migration, such as inflammatory diseases, tumor spread and metastases, without affecting other functions regulated by FPR1.

## Methods

### Molecular Modeling of FPR1

Human FPR1 (Uniprot code: P21462) was modelled using an integrated modeling approach of comparative modelling and folding *ab-initio*. In detail, we modeled the N-terminal domain (region 1-27)and the trans-membrane region (region 28-305), composed by seven helices and by three extracellular and three cytoplasmic loops, by comparative modeling using as template the structure of type-1 angiotensin II receptor (AGTR1, PDB code: 4YAY, UniProt code: P30556)^48^ and MODELLER program^49,50^. The C-terminal region (region 306-350)was modeled by Quark Server^37^. The best 3D models were selected on the basis of the energetic and stereo-chemical quality using ProSA program^51^ and Ramachandran plot^52^. Then, we have modeled the complete structure of FPR1 using as template the two structures obtained for N-terminal domain and the trans-membrane region and for C-terminal domain by MODELLER program^49,50^ as already reported in our recent paper^38^. FPR1 model has been deposited on Model Archive database and is accessible by the following link: 10.5452/ma-thpfw.

### Molecular modeling and dynamics of peptides

SRSRY and fMLF peptides were built by Builder module in Insight II and subjected to molecular dynamics (MD) simulations to study their energetic stability using GROMACS program and OPLS-AA force field^53^. Each peptide was inserted in a cubic box that contains water molecules and subjected to energy minimization and position restraints cycles. Then, MD simulations were performed for 100 ns at room temperature (300 K) by adding two chloride ions in the case of SRSRY to neutralize the net electrostatic charge of the system, as already published in the paper in which MD simulations on RI-3 are reported^33^. The trajectories were analyzed in terms of root mean squared deviation (RMSD), gyration radius fluctuations, root mean squared fluctuation (RMSF), secondary structure evolution and number of H-bonds using GROMACS routine.

### Molecular docking

The complexes between FPR1 and three peptides (SRSRY, fMLF and RI-3) were modeled by molecular docking studies using Patchdock algorithm based on three major stages: molecular shape representation, surface patch matching and filtering/scoring^54^. In detail, we used our complete obtained model for FPR1 and the final conformations obtained after MD simulations for SRSRY, fMLF and RI-3 peptides. The selection of the best complexes was made by visual inspection, discarding models in which the peptides were not located in the binding groove, as reported in literature^44,55,56^, and selecting the complexes with the highest number of H-bonds and salt bridges by HBPLUS^57^ and ESBRI^58^ programs and the highest number of amino acids by LigPlot^59^.

### Residue interaction network

For each FPR1-peptide complex, we analyzed the residue interaction network in which the amino acids are considered as nodes whereas the interactions between the residues as edges. In detail, Protein Interactions Calculator (PIC)^60^, HBPLUS^57^ and COCOMAPS^61^ tools were used to evaluate H-bonds, π-cations, π-stacking and IAC, respectively, that were inserted as interactions between the residues in the networks of FPR1-peptide complexes.

### Peptide synthesis

The SRSRY, ARARY and Ac-(D)-Tyr-(D)-Arg-Aib-(D)-Arg-NH_2_ (RI-3) peptides were custom-synthesized by JPT Peptide Technologies, Germany. The fluorescein-conjugate Ac-(D)-Tyr-(D)-Arg-Aib-(D)-Arg-Ahx-Lys (N^ɛ^-FITC)-NH_2_ (FITC-RI-3) peptide was custom-synthesized by MicroGem, Italy. These peptides were synthesized on solid-phase with Fmoc/t-Bu chemistry, purified by reversed-phase HPLC using water/acetonitrile gradients, and characterized by UPLC-MS. N-Formyl-L-methionyl-L-leucyl-L-phenylalanine (fMLF) and the fluorescein-conjugated hexapeptide formyl-Nle-Leu-Phe-Nle-Tyr-Lys (FITC-fMLF) peptides were purchased by Invitrogen.

### Cell lines

Rat basophilic leukemia RBL-2H3 cells which do not express FPR1 and RBL-2H3/ETFR cells stably transfected with human FPR1 cDNA^62^ were kindly provided by F. Blasi (IFOM, Milan, Italy). Human embryonic kidney HEK-293 cells which do not express uPAR and HEK-293 cells stably transfected with expression vector pcDNA3-uPAR (HEK-293/uPAR) have been previously described^13,16^. All cell lines were grown adherent in Dulbecco Modified Eagle Medium (DMEM) containing 10% Fetal Bovine Serum supplemented with 100 IU/mL penicillin and 50 μg/mL streptomycin and maintained in an atmosphere of humidified air with 5% CO_2_ at 37 °C.

### Cell migration

Cell migration in Boyden chambers was carried out as described^13,33,34^. Briefly, cell suspension (1 × 10^5^ viable cells/mL serum free medium) was seeded in each upper chamber. A subset of experiments were performed using cells desensitized with 100 nM fMLF or 100 nM SRSRY for 30 min at 37 °C in humidified air with 5% CO_2_ as described^14^. Lower chambers were filled with serum free DMEM containing diluent or the indicated peptides. The two compartments were separated by 8 μm pore size polycarbonate filters (Neuroprobe). Cells were allowed to migrate for 4 hr at 37 °C, 5% CO_2_. At the end of each assay, cells on the lower filter surface were fixed with ethanol, stained with haematoxylin and 10 random fields/filter were counted at 200x magnification. Each experiment was performed three times in triplicate.

### Ligand binding assay

Cells (1.5 × 10^6^ cells/sample) were pre-incubated with diluents (CTRL), 1 μM fMLF, 1 μM SRSRY, 1 μM ARARY, 1 μM RI-3 or increasing concentration of RI-3 for 30 min at 4 °C, extensively rinsed with phosphate buffer saline (PBS), exposed to 10 nM FITC-RI-3 diluted in binding buffer (PBS containing 25 mM Hepes), for additional 45 min at 4 °C and again rinsed with PBS. Then, cells were visualized with the fluorescent Axiovert 200 microscope (Carl Zeiss). Quantification of cell-associated fluorescence was assessed by reading cells with a fluorescence plate reader Victor 3 (Perkin Elmer) using 485 nm excitation and 535 nm emission filters as already reported^33,34^. The experiments were performed three times in triplicate.

### Fluorescence microscopy

Cells grown adherent on glass slides to semi-confluence were exposed to 10 nM FITC-RI-3 or 10 nM FITC-fMLF in PBS for 30 min at 37 °C and extensively rinsed with PBS. Then, coverslips were mounted using 20% (w/v) Mowiol, visualized with a Zeiss 510META-LSM microscope (Carl Zeiss), and z-series were collected at 0.19 µm intervals as reported^33,34^.

### Statistical analysis

Data from cell migration and binding assays were analyzed for significance using the Student’s t-test.

### Ethics statement

All experimental protocols were performed in accordance with guidelines of the Istituto Nazionale Tumori IRCCS “Fondazione G. Pascale” (Quality System n. LRC 6019486/QMS/U/IT- 2015 certificated in conformity with UNI EN ISO 9001:2008).

## Supplementary information

Supplementary Information

## Data Availability

All data generated or analysed during this study are included in this article and in the Supplementary Information file. Also, FPR1 model has been deposited on Model Archive database and is accessible by the following link: 10.5452/ma-thpfw.
