# Supplementary material for: Structure-function relationship of an Urokinase Receptor-derived peptide which inhibits the Formyl Peptide Receptor type 1 activity
Source: Sci Rep. 2019 Aug 21;9:12169. doi: 10.1038/s41598-019-47900-3 (PMC6704176; doi:10.1038/s41598-019-47900-3)
Supplement: Supplementary file 1 — Supplementary Information [file 41598_2019_47900_MOESM1_ESM.pdf]

Structure-function relationship of an Urokinase Receptor-derived peptide which inhibits the Formyl Peptide Receptor type 1 activity.

List of investigators: Michele Minopoli, Andrea Polo, Concetta Ragone, Vincenzo Ingangi, Gennaro Ciliberto, Antonello Pessi, Sabrina Sarno, Alfredo Budillon, Susan Costantini and Maria Vincenza Carriero.

Table of contents:

Supplementary Figures:

Supplementary Figure S1. Alignment of FPR1 and AGTR1 sequences used in the modeling procedure. Amino acids in the seven helices or in b-strands are evidenced in red and yellow, respectively. We inserted the numbering of FPR1 sequence in the alignment and evidenced the key residues of the binding pocket by a star (*).

Supplementary Figure S2. MD simulation of fMLF peptide: (a) RMSD plot, (b) RMSF plot, (c) gyration radius plot, (d) superimposition of MD frames at 0, 20, 60, 80 and 100 ns of fMLF peptide.

Supplementary Figure S3. MD simulation of SRSRY peptide: (a) RMSD plot, (b) RMSF plot, (c) gyration radius plot, (d) superimposition of MD frames at 0, 20, 60, 80 and 100 ns of SRSRY peptide.

Supplementary Figure S4. RBL-2H3/ETFR cells exposed to 10 nM FITC-RI-3 for 30 min at 37°C and visualized by a confocal microscope. Z-series images represent a subset of focal planes corresponding to 0.29 μm vertical interval of cells. Original magnification: 630x.

Supplementary Figure S5. RBL-2H3/ETFR cells exposed to 10 nM FITC-fMLF for 30 min at 37°C and then visualized by a confocal microscope. Z-series images represent a subset of focal planes corresponding to 0.29 μm vertical interval of cells. Original magnification: 630x.

Supplementary Figure S6. Binding properties of RI-3 to uPAR expressing cells. (a) HEK-293 and HEK-293/uPAR cells (1.5 x10^6 cells/sample) were pre-incubated with diluents (None), 1 μM SRSRY or 1 μM RI-3 for 30 min at 4°C and then exposed to 10 nM FITC-RI-3 for additional 45 min at 4°C. Fluorometric measurement of cell-associated fluorescence was assed using 485 nm excitation and 535 nm emission filters. Data are expressed as a percentage of the basal fluorescence intensity, normalized to 100% (CTRL) and represent a mean ± SD from two independent experiments, performed in duplicate. *Statistical significance against None with *$p<0.01$. (b). Images of HEK-293 and HEK-293/uPAR cells exposed to 10 nM FITC-RI-3 for 45 min at 4°C and then visualized using a Zeiss 510 Meta LSM microscope. Original magnification: 630x (c). Images of HEK-293/uPAR cells grown adherent on glass slides to semi-confluence, exposed to 10 nM FITC-fMLF or 10 nM FITC-RI-3 for 30 min at 37°C and then visualized using a Zeiss 510 Meta LSM microscope in 3D projection. Original magnification: 630x.

Supplementary Figure S1. Alignment of the FPR1 and AGTR1 sequences used in the modeling procedure.

```
               10        20        30        40        50
                |         |         |         |         |
FPR1   METNSSLPTNISGGTPAVSAGYLFLDIITYLVFAVTFVLGVLGNGLVIWVAGFRMT-HTV
AGTR1  ---------ILNSSDCPKAGRHNYIFVMIPTLYSIIFVVGIFGNSLVVIVIYFYMKLKTV

      60        70        80        90       100       110
       |         |         |*   **   |*        |**    *   |
FPR1   TTISYLNLAVADFCFTSTLPFFMVRKAMGGHWPFGWFLCKFVFTIVDINLFGSVFLIALI
AGTR1  ASVFLLNLALADLCFLLTLPLWAVYTAMEYRWPFGNYLCKIASASVSFNLYASVFLLTCL

     120       130       140       150       160        170
       |         |         |         |         |          |
FPR1   ALDRCVCVLHPVWTQNHRTVSLAKKVIIGPWVMALLLTLPVIIRVTTV-PGKTGTVACTF
AGTR1  SIDRYLAIVHPMKSRLRRTMLVAKVTCIIIWLLAGLASLPAIIHRNVFFIENTNITVCAF

      180       190       200       210       220       230
        |         |         |*   *    |         |         |
FPR1   NFSPWTNDPKERINVAVAMLTVRGIIRFIIGFSAPMSIVAVSYGLIATKIHKQGLIKSSR
AGTR1  HYESQNSTL----------PIGLGLTKNILGFLFPFLIILTSYTLIWKALKKAYEIQKNK

        240       250       260       270            280       290
          |         |   *  *  |         |              |   *  *  |
FPR1   P--LRVLSFVAAAFFLCWSPYQVVALIATVRIRELL-----QGMYKEIGIAVDVTSALAF
AGTR1  PRNDDIFKIIMAIVLFFFFSWIPHQIFTFLDVLIQLGIIRDCRIADIVDTAMPITICIAY

             300
               |
FPR1   FNSCLNPMLYVFMGQ
AGTR1  FNNCLNPLFYGFLGK
```

Amino acids in the seven helices or in b-strands are evidenced in red and yellow, respectively. We inserted the numbering of FPR1 sequence in the alignment and evidenced the key residues of the binding pocket by a star (*).

Amino acids in the seven helices or in ß-strands are highlighted in red and yellow, respectively.

Supplementary Figure S2. MD simulation of the fMLF peptide.

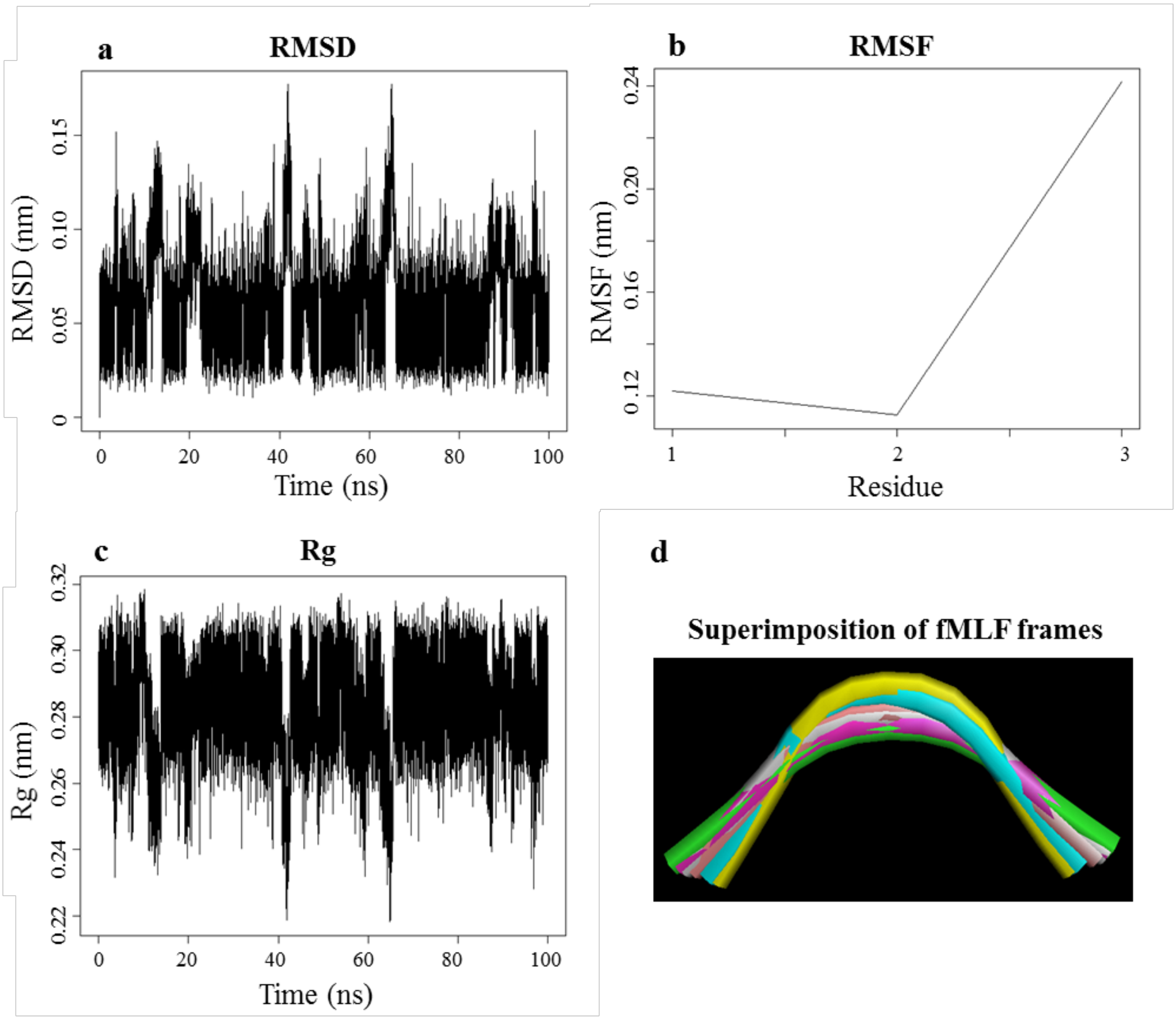

(a) RMSD plot, (b) RMSF plot, (c) gyration radius plot, (d) superimposition of MD frames at 0, 20, 60, 80 and 100 ns of fMLF peptide.

Supplementary Figure S3. MD simulation of SRSRY peptide.

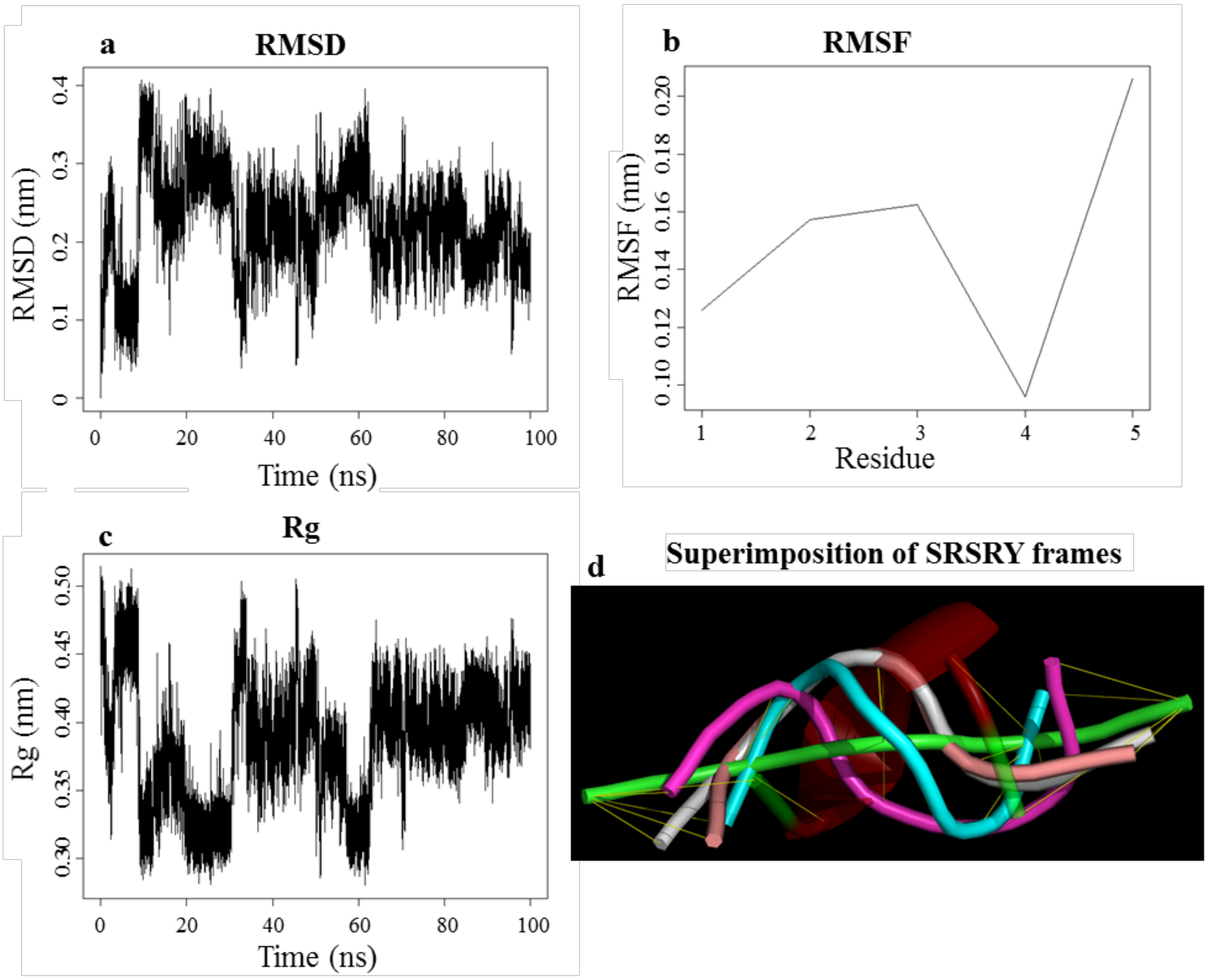

(a) RMSD plot, (b) RMSF plot, (c) gyration radius plot, (d) superimposition of MD frames at 0, 20, 60, 80 and 100 ns of SRSRY peptide.

Supplementary Figure S4. Internalization of the FITC-RI-3 peptide.

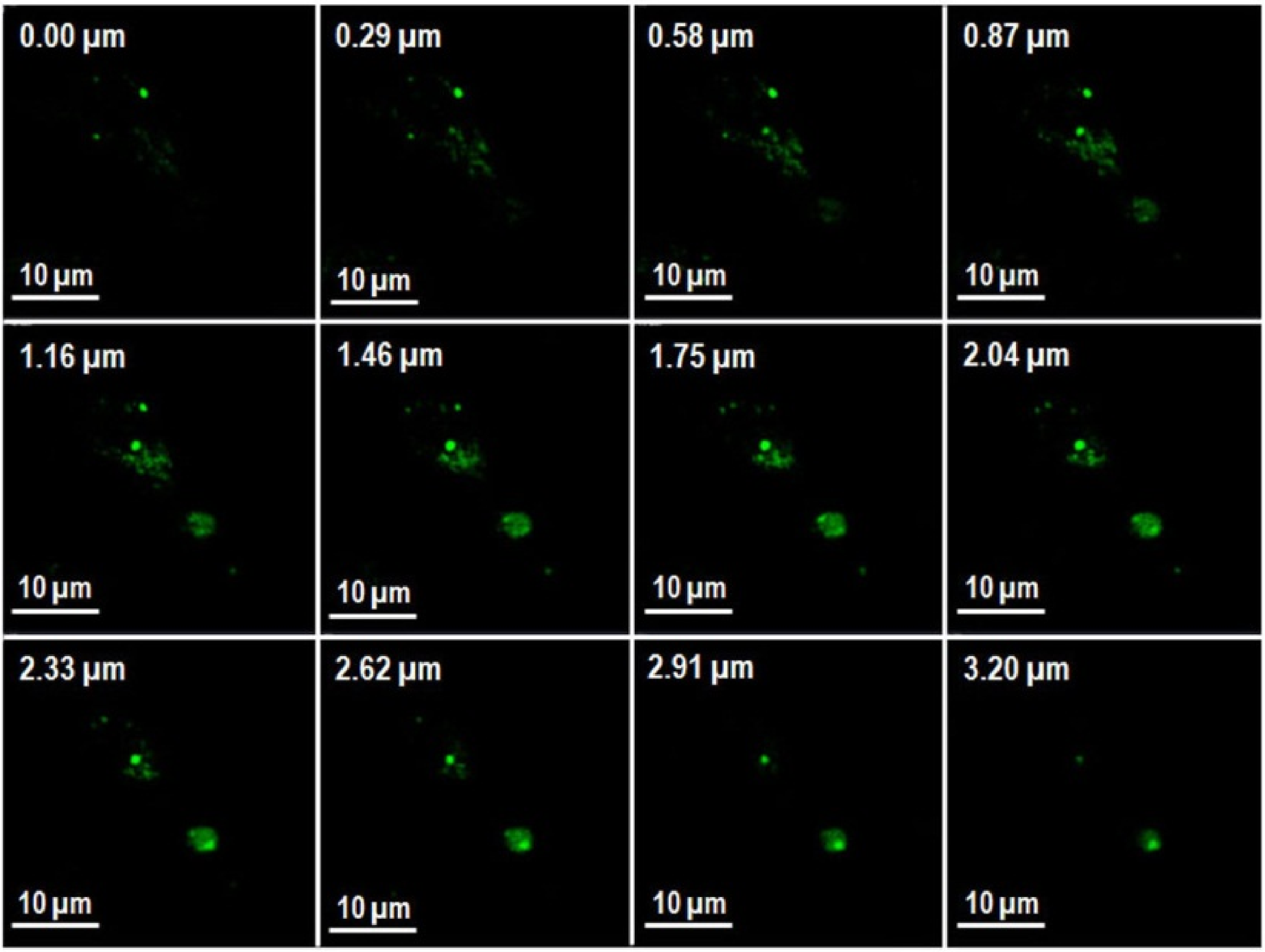

RBL-2H3/ETFR cells exposed to 10 nM FITC-RI-3 for 30 min at 37°C and visualized by a confocal microscope. Z-series images represent a subset of focal planes corresponding to 0.29 µm vertical interval of cells. Original magnification: 630x.

Supplementary Figure S5. Internalization of the FITC-fMLF peptide.

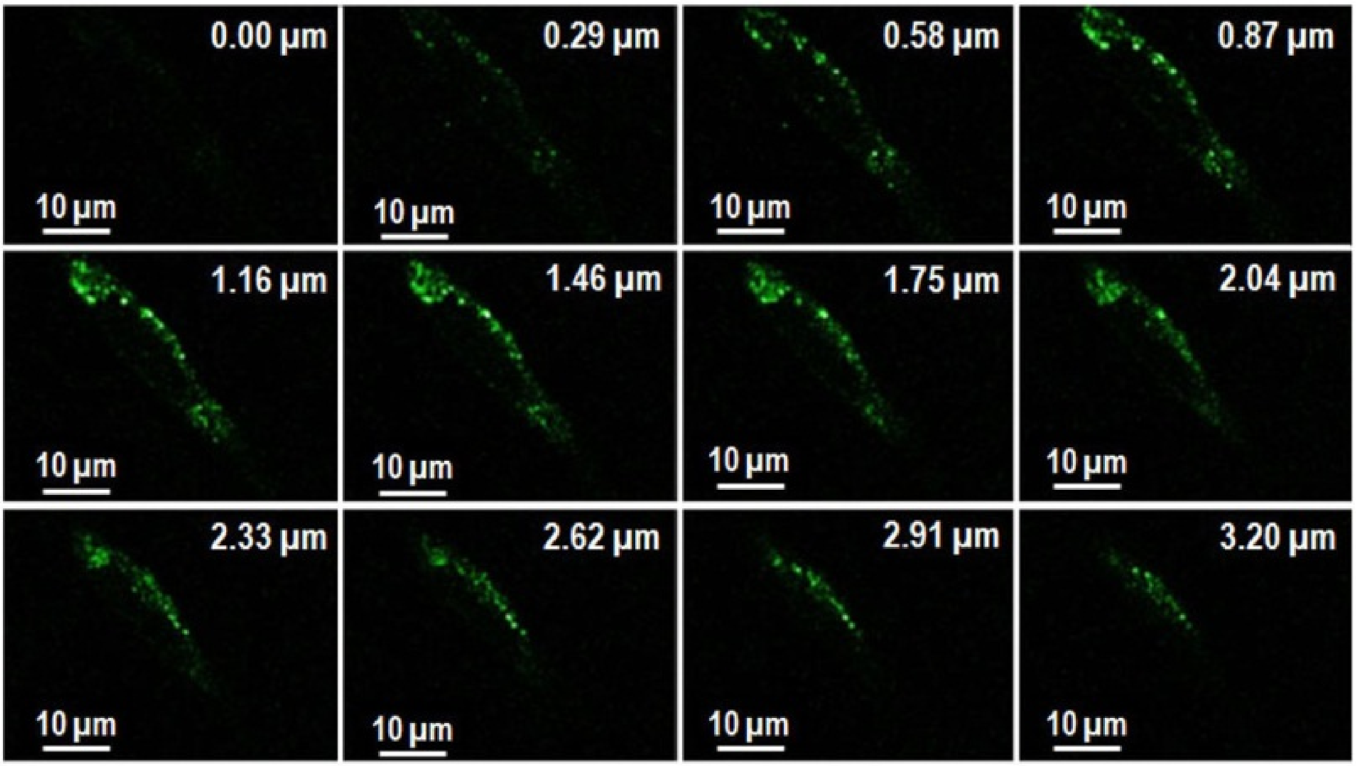

RBL-2H3/ETFR cells exposed to 10 nM FITC-fMLF for 30 min at 37°C and then visualized by a confocal microscope. Z-series images represent a subset of focal planes corresponding to 0.29 µm vertical interval of cells. Originalmagnification: 630x.

Supplementary Figure S6. Binding properties of RI-3 to uPAR expressing cells.

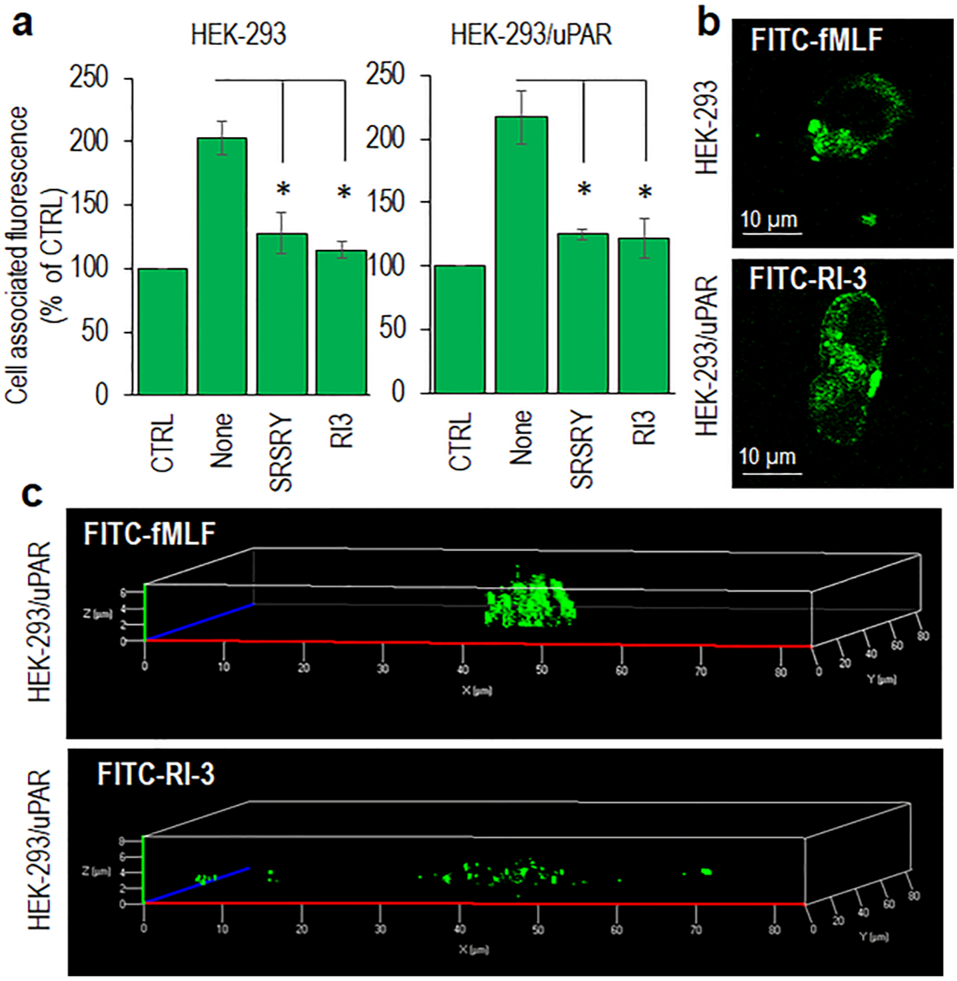

(a) HEK-293 and HEK-293/uPAR cells (1.5 x10^6 cells/sample) were pre-incubated with diluents (None), 1 µM SRSRY or 1 µM RI-3 for 30 min at 4°C and then exposed to 10 nM FITC-RI-3 for additional 45 min at 4°C. Fluorometric measurement of cell-associated fluorescence was assed using 485 nm excitation and 535 nm emission filters. Data are expressed as a percentage of the basal fluorescence intensity, normalized to 100% (CTRL) and represent a mean ± SD from two independent experiments, performed in duplicate. *Statistical significance against None with $*p<0.01$. **(b).** Images of HEK-293 and HEK-293/uPAR cells exposed to 10 nM FITC-RI-3 for 45 min at 4°C and then visualized using a Zeiss 510 Meta LSM microscope. Original magnification: 630x **(c).** Images of HEK-293/uPAR cells grown adherent on glass slides to semi-confluence, exposed to 10 nM FITC-fMLF or 10 nM FITC-RI-3 for 30 min at 37°C and then visualized using a Zeiss 510 Meta LSM microscope in 3D projection. Original magnification: 630x.
